# Imaging of the scrotum: beyond sonography

**DOI:** 10.1007/s13244-017-0592-z

**Published:** 2018-02-15

**Authors:** Gian Carlo Parenti, Francesco Feletti, Aldo Carnevale, Licia Uccelli, Melchiore Giganti

**Affiliations:** 1 Department of Diagnostic Imaging of Romagna, Section of Radiology, Ospedale Civile Santa Maria delle Croci, 48100 Ravenna, Italy; 2 Department of Morphology, Surgery and Experimental Medicine, Section of Radiology, University of Ferrara, Via Ludovico Ariosto 35, 44121 Ferrara, Italy; 3 Department of Morphology, Surgery and Experimental Medicine, Section of Nuclear Medicine, University of Ferrara, Via Ludovico Ariosto 35, 44121 Ferrara, Italy

**Keywords:** Magnetic resonance imaging, Scrotum, Testis, Ultrasonography, Male urogenital diseases

## Abstract

**Abstract:**

The aim of this article is to describe the role of second-level imaging techniques after an initial ultrasonography evaluation in the assessment of scrotal diseases. While ultrasonography remains central as the primary imaging modality for the evaluation of pathologic conditions of the scrotum, the role of magnetic resonance imaging continues to evolve: it can actually be valuable as a problem-solving tool when sonographic findings are equivocal or inconclusive. Magnetic resonance imaging of the scrotum may provide accurate detection and characterization of scrotal diseases, well depicting the precise location of scrotal masses (intratesticular or extratesticular) and reliably characterizing benign conditions simulating neoplastic processes, thus preventing unnecessary radical surgery. Advanced magnetic resonance techniques, most of all diffusion weighted imaging and magnetic resonance spectroscopy, play in the meanwhile a more significant role in evaluating scrotal diseases.

**Teaching points:**

*• Multiparametric ultrasonography usually represents the initial imaging modality for approaching scrotal diseases.*

*• MRI is well established as a problem-solving tool for inconclusive sonographic findings.*

*• Advanced MRI techniques can be successfully applied in scrotal pathology assessment.*

*• MRI is valuable in differentiating benign conditions from neoplastic processes.*

*• CT plays a role in trauma assessment and cancer staging alongside PET/CT.*

## Introduction

Multiparametric ultrasonography (US), encompassing in this definition conventional Brightness-Mode US, Eco-colour Doppler analysis, contrast-enhanced ultrasonography (CEUS) and elastosonography, usually represents the initial imaging modality for approaching scrotal diseases, thanks to a proven diagnostic accuracy, cost-effectiveness and large availability of this technique [1–3].

Nevertheless, US correlates to some important limitations: the operator-dependent nature of the modality, low panoramicity due to small field of view and poor tissue characterization [1, 4].

Therefore, other imaging modalities could integrate ultrasound examination, in order to clarify any inconclusive or equivocal finding, or with the purpose of longitudinal monitoring scrotal disease.

In the literature, the role of MRI is well established as a second level imaging modality useful for an increasing number of indications [5–8].

Advanced techniques, namely diffusion weighted imaging (DWI), proton magnetic resonance spectroscopy (H1MRS) and magnetization transfer ratio (MTR), applied successfully in the evaluation of other anatomical districts, could be also used as an adjunctive imaging tool to assess testicular pathology [9–15].

The aim of this article is to describe the role of second-level imaging modalities, after ultrasonography examination, in the assessment of scrotal diseases.

## Materials and methods

An extended systematic search in Medline database (via PubMed), including articles related to human medicine published in the last 10 years to January 1, 2017, was performed. Keywords were chosen according to Medical Subject Heading (MeSH) terms: (“Diagnostic Imaging”[Mesh]) AND (“Testis”[Mesh]) OR (“Scrotum”[Mesh]) NOT “Ultrasonography”[Mesh].

The bibliographic search produced 443 results; two independent reviewers (G.P. and F.F. with more than 25 and 11 years of experience in urogenital radiology, respectively) screened the initial search results on the basis of titles and abstracts. Both reviewers had experience in data extraction for retrospective and prospective studies.

Articles addressing an issue pertinent to magnetic resonance imaging (MRI), computed tomography (CT) or positron emission tomography (PET)-CT examination of scrotal diseases were considered potentially eligible for inclusion. Studies only focused on scrotal ultrasound were excluded. Because of the variety of the issues dealt with by this work and due to limited data available from the literature, the reviewers included case reports considered to contribute relevant information about imaging techniques application or for future research. All the papers not included in the first literature selection of each of the two reviewers, were jointly re-analysed and discussed to obtain an agreement.

The full articles of selected studies were retrieved and additional searches of their reference lists performed to identify other potentially eligible articles. All non-English written studies were evaluated only by the information taken from the abstract.

In order to aid the selection procedure, a score was assigned to each article that met the inclusion criteria according to the 2011 Oxford Center of Evidence-Based Medicine (CEBM) levels of evidence [16], as follows:

1. systematic review of cross sectional studies with consistently applied reference standard and blinding;
2. individual cross sectional studies with consistently applied reference standard and blinding;
3. non-consecutive studies, or studies without consistently applied reference standards;
4. case-control studies, or poor or non-independent reference standard;
5. mechanism-based reasoning.

Finally, 58 articles were considered relevant for the scopes of the literature review and thus included in the present work.

## Results

MRI, thanks to high spatial resolution and a large field of view, can be considered an ideal second-level technique [4], useful as a problem-solving tool when sonographic findings are equivocal [6].

We already found in a previously published study [7] that MRI proved to be necessary in 5.74% of patients (tot. *n* = 801) suspected to have scrotal disease after sonographic evaluation. In 47.8% of the cases, MRI managed to achieve a correct diagnosis, while in 37% of the cases contributed to exclude focal lesions. As Muglia et al. [8] reported in their work, also in our experience MRI was necessary in 5.02% of patients (tot. *n* = 622) undergone US, and resulted to add specific value in 82% of the cases.

In addition to MRI, Computed Tomography (CT) and positron emission tomography-CT (PET-CT) have a second-level role approaching neoplastic processes, as shown below.

This review is organized on a pathology basis, as follows: trauma, inflammatory pathology, tumours, infarct, varicocele and infertility functional assessment, congenital abnormalities and scrotal hernia.

### Trauma

Kim et al. [17] proved a 100% diagnostic accuracy for MRI in blunt scrotal trauma (7/7 cases). More in detail, authors reported two doubtful cases on sonographic evaluation for which MRI managed to achieve a diagnosis of epididymal hematoma.

In all cases of trauma (4) reported by Muglia et al., MRI identified a testicular hematoma or hematocele, assessing its extension and eventually inguinal canal involvement [8].

In our experience, in three patients with suspected neoplastic lesion according to US findings, MRI was useful in proving a testicular hematoma leading to a conservative approach. Furthermore, in a case it revealed an intrascrotal tear of tunica albuginea surrounding corpus cavernosum [7, 18] not noted on US examination.

CT provides excellent depiction of bone fragments, defining their presence and location, and eventual testicular dislocation resulting from a blunt pelvic injury [19–21].

### Inflammatory processes

In our series, we described a case of epididymo-orchitis evolved into abscess formation, complicated by an intra-scrotal fistula poorly characterized by sonography; in that occasion, particularly thanks to T2-weighted sequences, MRI allowed identifying fistulous track and its relationship to close structures [7]. We also reported three cases of severely inhomogeneous testicular echogenicity, in which MRI allowed excluding focal signal intensity abnormalities or focal areas of pathologic contrast-enhancement (CE), differentiating between a chronic orchitis from a neoplastic process, thus preventing unnecessary surgery [7].

In the literature, nevertheless we found cases of chronic granulomatous orchitis confused with tumour and undergone orchiectomy on the basis of MRI findings of testicular enlargement, hypointensity on T2-weighted images and increased signal on DW images [22].

Finally, MRI utility in early diagnosis of Fournier gangrene and in pre-operative planning is illustrated in a few works, accurately depicting fascial spread of the inflammatory process and necrotic component [23].

### Tumours

In a study conducted on 165 testicle-containing scrotal compartments (84 patients), MRI proved 100% sensitivity, specificity, positive predictive value and negative predictive value for precise localization of scrotal focal lesions, excellently differentiating testicular from extra-testicular ones [24].

Muglia et al. found that MRI allowed reliable characterization of testicular tumours and pseudotumors in 88% of the cases (*n* = 15); findings not recognized at US that were essential to define the neoplastic nature of the process were the presence of hemorrhagic intralesional components and extension to tunica albuginea or epididymis [8]. Nevertheless, in the same series, two lesions suspected for tumours at MRI revealed chronic orchitis at pathological exam after surgery.

Some reports [25, 26] described hypointensity signal on T2-weighted images and lack of restricted diffusion on DWI as useful in discriminate between paratesticular fibrous pseudotumor and malignancies, clearly affecting patient management avoiding radical surgical intervention.

In our experience, MRI had an essential role in 15 cases of testicular neoplasms: in 11 cases of malignant germ cell tumours and 4 cases of benign lesions, of which two were Sertoli cell tumours and two were adenomatoid tumours. In all these cases, MRI was useful for preoperative planning, providing excellent depiction of eventual infiltration of tunica albuginea, tunica vaginalis, epididymis and paraepididymal soft tissues [7].

In a study conducted on 33 cases of testicular masses detected on US, Tsili et al. suggested that sensitivity and specificity of MRI in differentiating benign from malignant lesions was 100% (95% CI, 87.9–100%) and 87.5% (95% CI, 52.9–97.7%), respectively, with an overall accuracy of 96.4% and a satisfactory rate of correspondence between MRI findings and histopathologic diagnosis in regard to local extension of testicular neoplasms (92.8%) [27]. All malignant tumours exhibited mainly hypointense signal compared to contralateral normal testis on T1-weighted images, whereas signal intensity on T2-weighted images was low in seminomas, showing intralesional septal contrast enhancement after gadolinium administration. Non-seminomatous tumours were heterogeneous on both T1 and T2-weighted images, containing cystic or necrotic components, with heterogeneous enhancement [27]. In the same study, investigators also described a hypointense testicular focal lesion with contrast enhancement, falsely suspected for malignancy but revealed to be granulomatous orchitis after surgical biopsy.

Manganaro et al., in their work concerning contrast enhanced MRI on 44 patients with nonpalpable testicular lesions, found in benign lesions a more rapid and intense wash-in (18/21, 85.7%, vs. 5/23, 21.7%) and a prolonged wash-out (15/21, 71.4% vs. 0/23), whereas malignant tumours showed a weak and progressive wash-in (18/23, 78.3% vs. 3/21, 14,3%) and an absent wash-out (21/23, 91.3% vs. 6/21, 28.6%) [28].

The role of DW imaging has been evaluated in a study conducted on 50 patients with monolateral testicular disease. A cut-off ADC value of ≤0.99 × 10^−3^ mm^2^/s had a sensitivity of 93.3%, specificity of 90%, positive predictive value of 87.5% and negative predictive value of 94.7% in the characterization of intratesticular masses [29]. Tsilli et al., in a series of 26 men, suggested that ADC values could be used to differentiate seminomas from non-seminomatous germ cells tumours before surgical intervention [30]. Moreover, a retrospective evaluation of 56 cases advocated that ADC values do not provide detection of Testicular Intraepithelial Neoplasia (TIN) and could not be considered as a non-invasive screening tool for patients requiring surgical biopsy [31].

In a meta-analysis on 130 patients, fluorine-18-fluorodeoxyglucose (FDG)-PET-CT proved to be the best imaging technique capable to detect residual disease in metastatic seminomas after chemotherapy, correctly directing therapeutic management [32]; FDG-PET demonstrated higher specificity (92% vs. 59%) and sensitivity (72% vs. 63%), as well as a higher positive (70% vs. 28%) and negative predictive value (93% vs. 86%) than the solely size-based CT assessment of residual tumours.

### Testicular infarction

In a previously published study [33], in 14 patients suspected for Segmental Testicular Infarction (STI) at US examination MRI allowed a correct diagnosis. In more detail, in 92% of the cases (*n* = 13) diagnosis was confirmed by imaging findings, i.e. hypointense signal on both T-1 and T2-weighted images, and lack of internal contrast enhancement of the infarcted tissue. In a single case, STI was excluded for the presence of a mild intralesional contrast enhancement and lack of change at close imaging follow-up; the patient underwent orchiectomy with a histopathology report of B-cells lymphoma.

Urgent MRI, including dynamic contrast enhanced (DCE)-MRI, has a 100% sensitivity in the diagnosis of testicular torsion, whereas T2- and T2*-weighted images have a 100% accuracy in detection of testicular necrosis [34].

### Varicocele and infertility functional assessment

After a sonographic diagnosis and evaluation of varicocele, magnetic resonance angiography (MRA) allows depiction of eventual associated conditions, e.g. nutcracker syndrome [35]. Similarly, antegrade venography may identify the level of a possible parallel duplication of internal spermatic vein, allowing an optimal treatment [36].

Karakas et al. [37] investigated the role of ADC values in determining testicular fibrosis; the authors found that ADC values were significantly lower among 25 patients presenting with varicocele compared to those of healthy volunteers; ADC values were also negatively correlated with venous diameter.

Also, in hydrocele evaluation, in a series of 49 patients, altered ADC values were reported consequentially to a mechanical effect of the pressure of the fluid, with possible dysfunction of testicular tissue and a negative effect on fertility [38].

To assess the utility of MR spectroscopy in the evaluation of male infertility, we have studied 14 patients affected with documented fertility dysfunction (five patients presenting with oligospermia, three with asthenospermia, six with oligo-asthenospermia). The choline principal peak was significantly lower in the altered semen analysis group than in normal controls (0.69 vs 1.34, 95% CI: 0.52–0.85; *p* < 0.001). Since patients proved to have semen alterations did not show any significant morphological finding in testicle structure both at US and MRI, excepted for varicocele in seven cases, we concluded that 1HMRS is capable to reveal spermatogenesis disorders in patients with normal testis at US and MRI examination [39].

### Congenital abnormalities and scrotal hernia

In a meta-analysis (8 studies) on 171 patients with 193 non-palpable testicles [40], investigators proved that conventional MRI has a low sensitivity (estimated median sensitivity of 62%), but high specificity (100%) in the identification of nonpalpable cryptorchid testes, and has limited value in locating intra-abdominal viable undescended testicles, whereas it fails in determining the presence of most atrophic ones. However, T2-weighted sequences with fat-suppression techniques, DWI [41] or MRA may significantly improve accuracy of MR in cryptorchidism assessment [40].

MRI could allow or confirm the diagnosis of transverse testicular ectopia and eventually show any other associated variants or abnormalities [42, 43]. In a case report of an accessory scrotum, Ziegelmann et al. found that MRI was capable to define the complex relationships between the accessory scrotum and ano-sphincteric complex, as well as identify associated peritoneal lipoma [44].

In our series, MRI confirmed a sonographic diagnosis of inguinal-scrotal hernia in three patients, demonstrating the presence of intestinal loops in hernia sac [7].

## Discussion

### Trauma

MRI in scrotal trauma assessment provides excellent depiction of hematomas based on the signal characteristics of blood products, i.e. hyperintense signal on T1-weighted images related to methaemoglobin, associated to hypointense on T2-weighted images perilesional hemosiderin rind.

On MRI, considering that testicular tumours are incidentally identified in 15% of patients after a traumatic episode, a confident differentiation between a hematoma and a neoplastic mass can be easily achieved in most cases, despite confusing US appearance of an inhomogeneous focal lesion, with intralesional vascular signals occasionally observed at colour Doppler analysis [45].

**Fig. 1:**
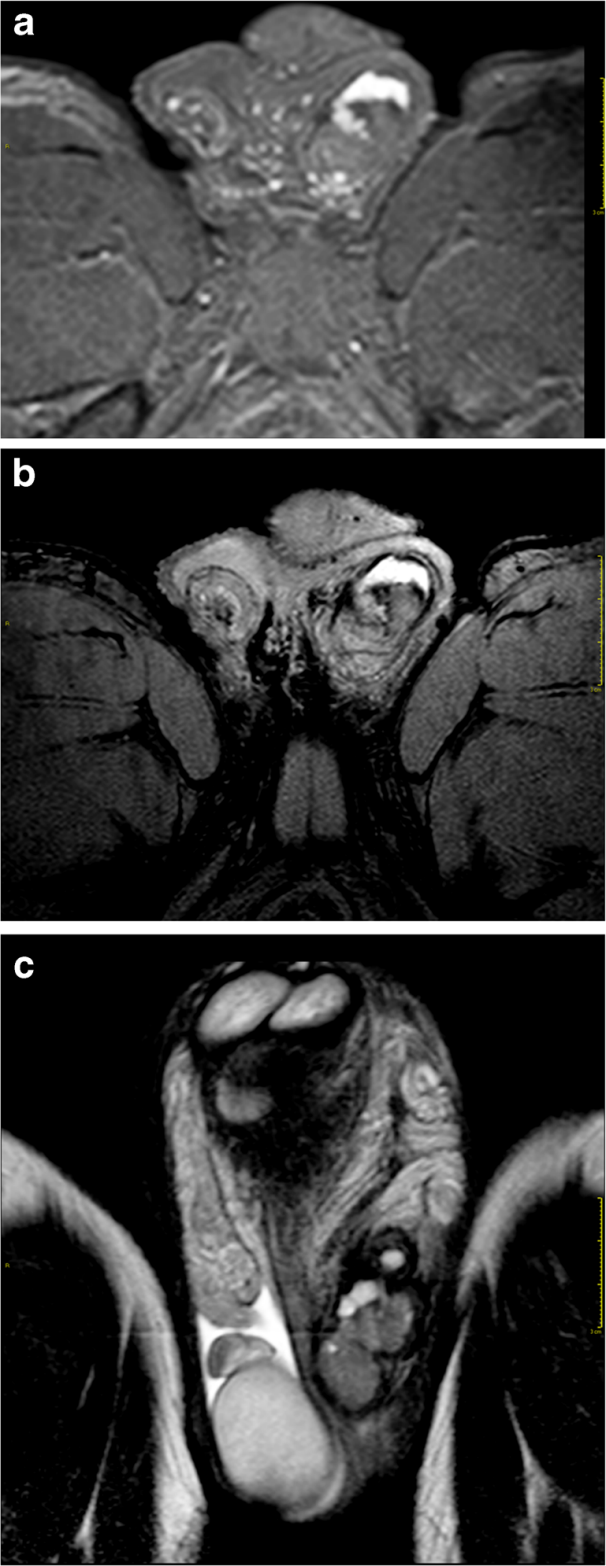
**a**-**c** Left testicle rupture. MRI shows a tunica albuginea tear and testicle rupture. **a** T1-weighted SPIR TSE sequence, axial view, **b** T2-weighted FFE sequence, axial view; **c** T2-weighted TSE sequence, coronal view

**Fig. 2:**
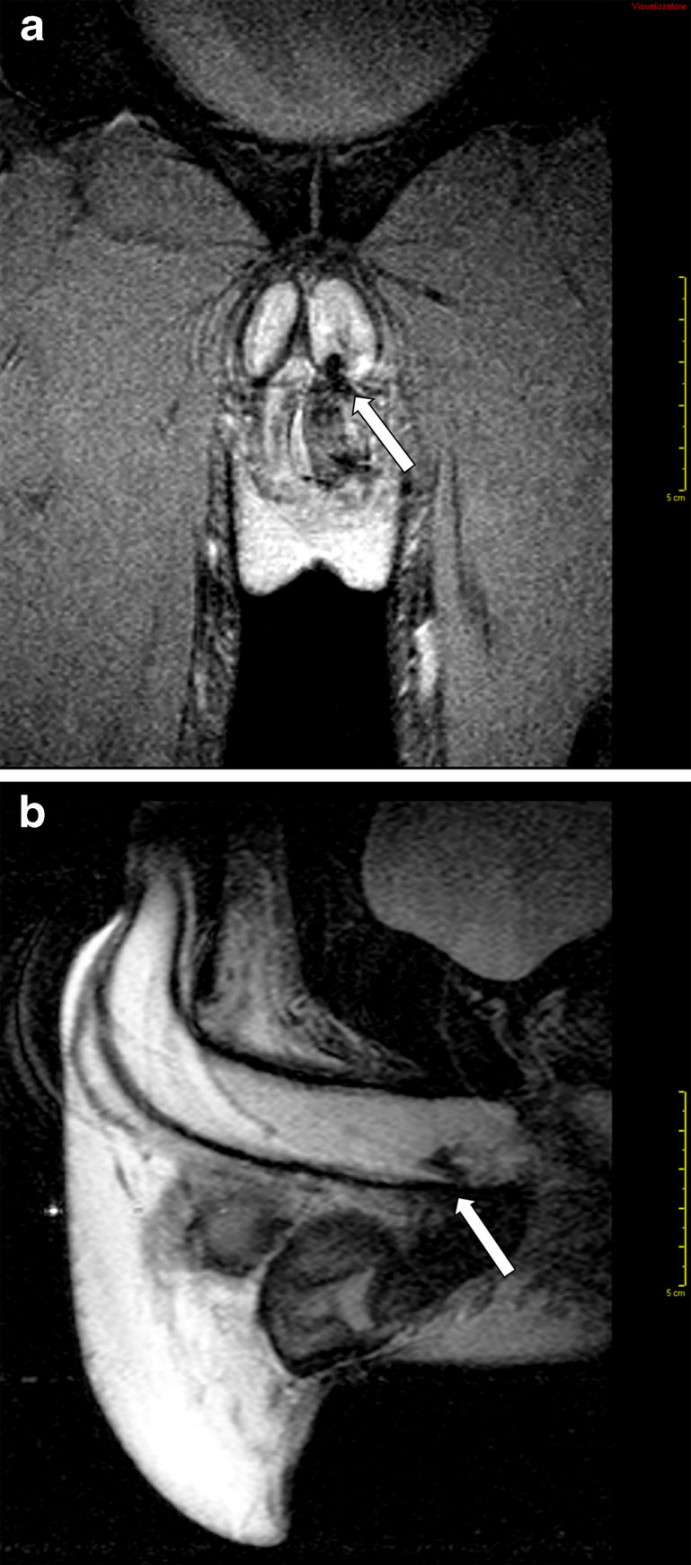
**a**-**b** Intrascrotal rupture of the corpus cavernosum (arrow). **a** T2-weighted FFE sequence, coronal view; **b** T2-weighted SPIR TSE sequence, sagittal view

Moreover, due to a large field of view and optimal soft tissue characterization, MRI not only defines the extension of traumatic testicular injuries previously identified on US (Fig. 1), but also demonstrates an associated tunica albuginea tear in corpora cavernosa, providing information in regard to the vascularization, fundamental for an optimal surgery planning (Fig. 2). On this regard, we consider MRI usage for monitoring results of conservative therapies when a tunica albuginea tear is recognized, but a normal capsular blood flow of the tunica vasculosa is preserved [7].

CT is crucial in the evaluation of complex pelvic traumas involving the scrotum, revealing the presence and precise location of bone fragments, as well as testicular dislocation [19–21].

### Inflammatory processes

MR imaging may prove useful in inflammatory scrotal conditions as an adjunct to US in defining the extent of inflammation, thereby aiding in the differential diagnosis from neoplasms. Actually, based only on imaging findings, inflammation if localized can mimic a neoplastic process [46]; in this setting, clinical correlation is usually decisive for orienting the differential diagnosis [27].

Acute orchitis usually manifests as an area of heterogeneous signal intensity, reduced on T1-weighted images and increased on T2, in association to epididymis swelling and scrotal layers thickening [47].

In granulomatous orchitis, the absence of areas of altered signal intensity and contrast enhancement can orientate the diagnosis [7], as well as a concomitant epididymal involvement [27]; nevertheless, a chronic orchitis can simulate a neoplasm despite using more advanced MR imaging techniques [6, 22, 27]. In these cases, biopsy [6] or MRI follow-up after medical treatment may prevent unnecessary orchiectomy.

Moreover, MRI can confirm the sonographic detection of a complicated testicular cyst, depicted as slightly hypoechoic on US and presenting as a well-marginated area of typical high signal intensity on T2-weighted images with also high signal intensity on T1-weighted images due to inflammatory content [7].

**Fig. 3:**
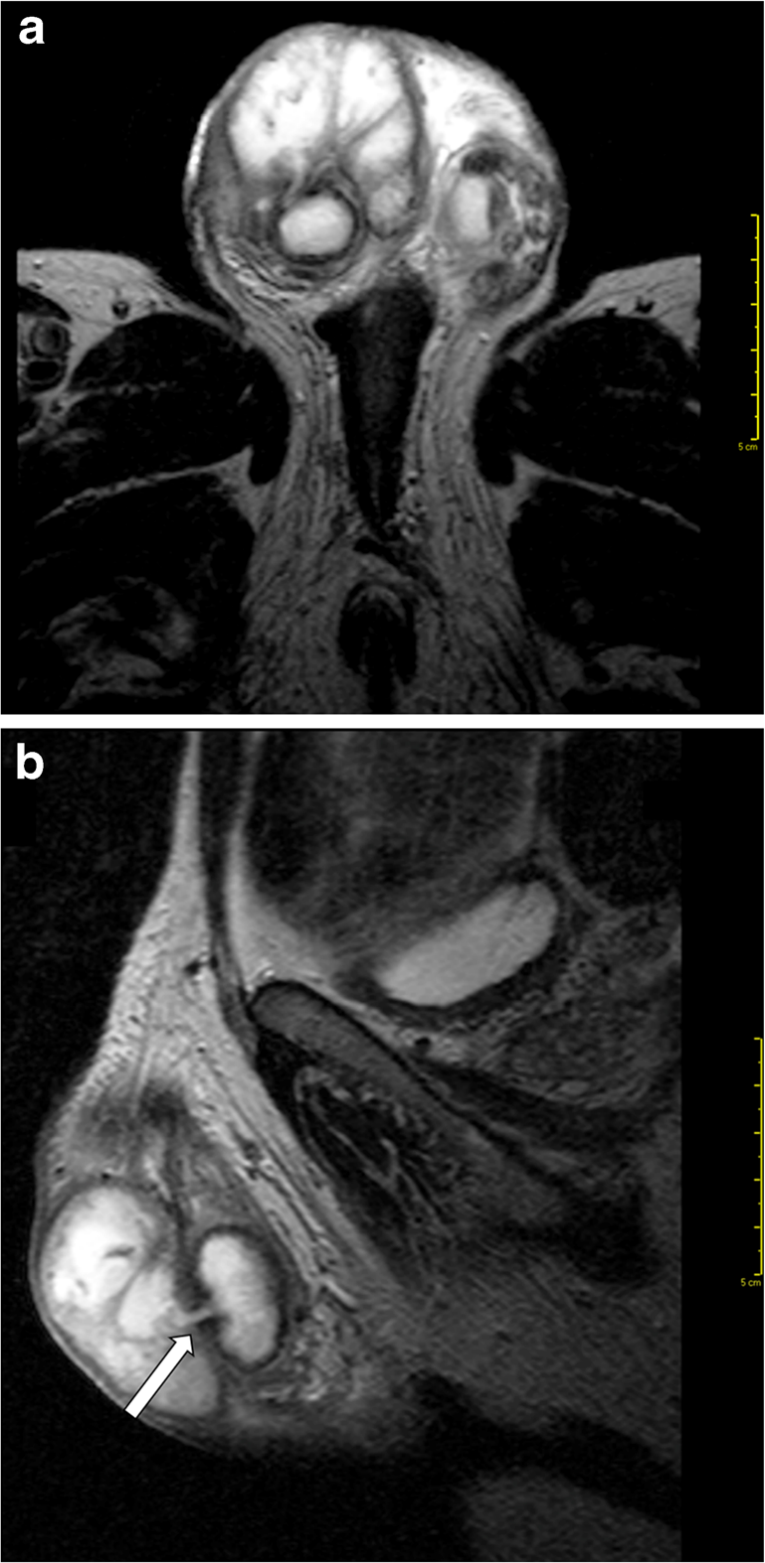
**a**-**b** Intrascrotal abscess. **a** T2-weighted TSE sequence, axial view, and **b** T2-weighted TSE sequence, sagittal view, showing fistulous track (arrow)

MRI is also helpful for the exact depiction of more conspicuous inflammatory processes, demonstrating extrascrotal tissues involvement of great intrascrotal abscesses [7]. T2-weighted sequences enable excellent depiction of fistulous tracks (Fig. 3).

### Tumours

**Fig. 4:**
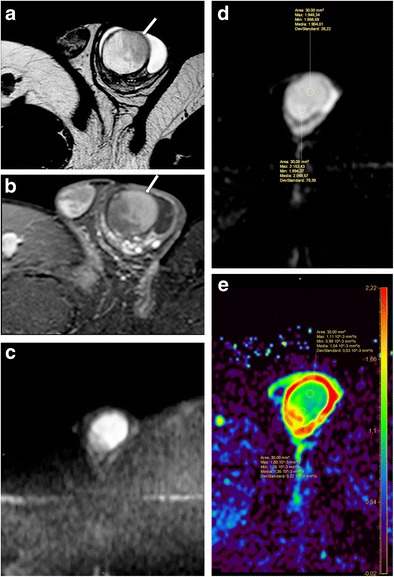
**a**-**e** MR characterization of post-traumatic fibrosis. **a** T2-weighted TSE sequence, axial view and **b** post-contrast T1-weighted SPIR TSE sequence, axial view, depicting an ill-defined hypointense on T2-weighted image (T2-wi) area, with mild contrast enhancement (arrow), consistent with fibrosis; **c** DWI, b = 800; **d**-**e** ADC map

**Fig. 5:**
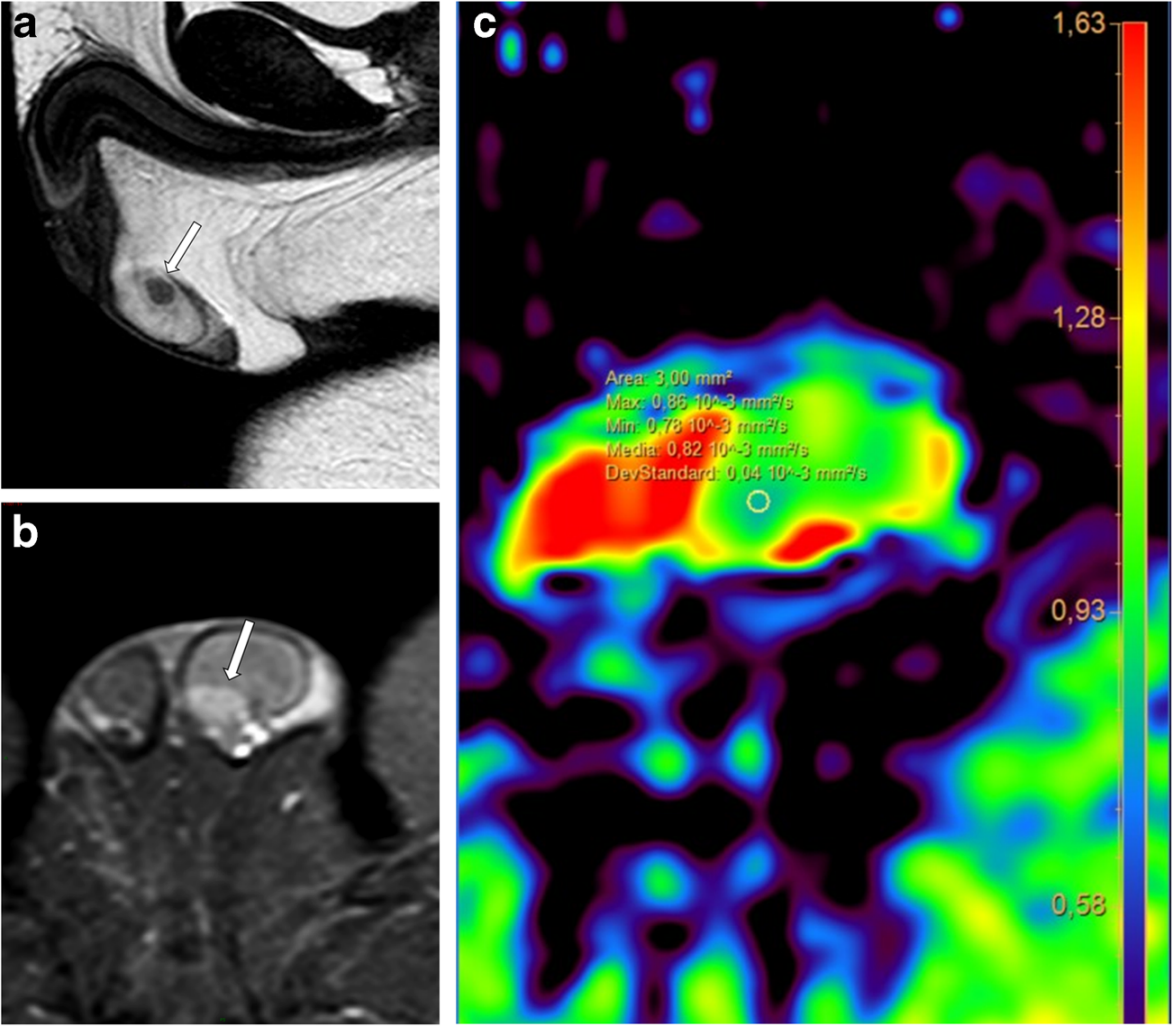
**a**-**c** MR study in a case of benign Leydig cells tumour. **a** T2-weighted TSE sequence, sagittal view and **b** post-contrast T1-weighted SPIR TSE sequence, axial view, depicting a round hypointense on T2-wi testicular focal lesion (arrow), with homogeneous hyperenhancement. **c** ADC map

**Fig. 6:**
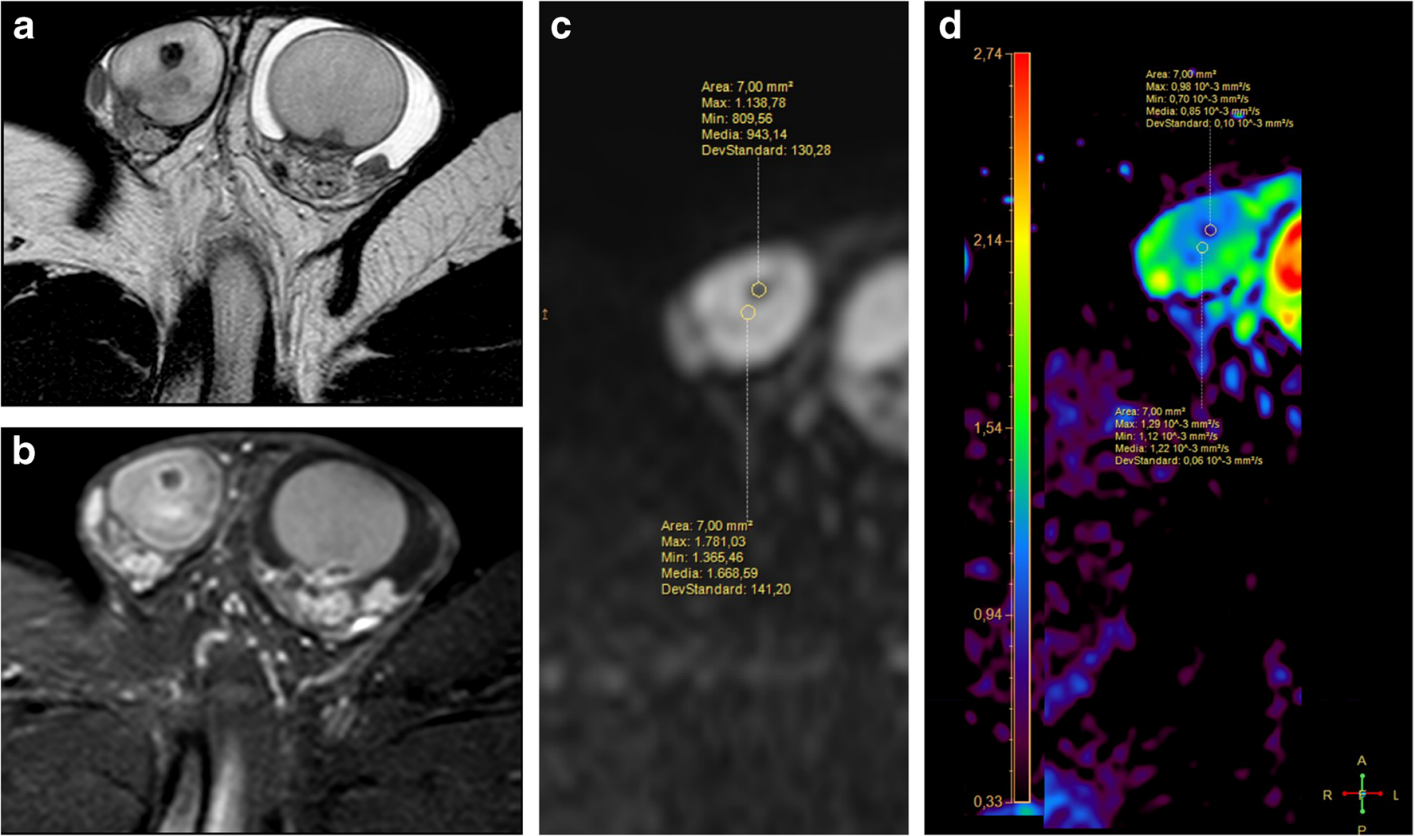
**a**-**d** MR study of a multifocal mixed embryonal carcinoma. **a** T2-weighted TSE sequence, axial view; **b** postcontrast T1-weighted SPIR TSE sequence.; **c**-**d** ADC

**Fig. 7:**
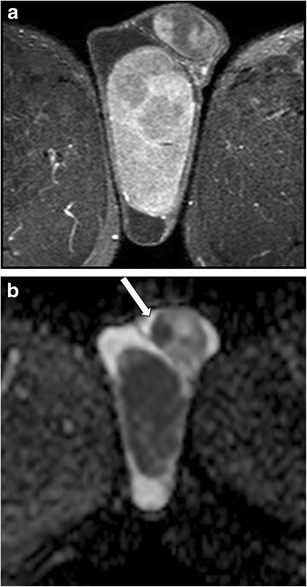
**a**-**b** MRI in a case of bilateral seminoma. **a** Post-contrast T1-weighted SPIR TSE sequence, axial view and **b** ADC map, demonstrating a large right testicular mass and a smaller left intratesticular one (arrow), with heterogeneous contrast enhancement and restricted diffusion

MRI is highly valuable in the characterization of testicular masses detected at US examination [7], notably of nonpalpable testicular lesions [28]. This technique actually enables an accurate differentiation of tumours from pseudotumors, namely chronic orchitis, post-traumatic fibrosis (Fig. 4) and rete testis ectasia [8], and, among tumours, allows identifying morphological features of malignancy (Fig. 5), for instance extratesticular structures involvement [8], or the presence of multifocal disease (Figs. 6 and 7), proving high rate of correspondence between imaging findings and histopathological diagnosis in regard to local extent [27].

Scrotal MRI performs well with respect to the differentiation of extratesticular from intra-testicular disease, and can aid in narrowing the differential diagnosis in cases of paratesticular masses [48]. Paratesticular sarcomas, that may involve the testicle itself [49], can mimic an inguinal hernia at clinical and sonographic evaluation; CT and MRI both confidently demonstrate the presence of fat component, calcifications, sepiments or intralesional contrast enhancement, helping discerning these tumours from hernias and spermatic cord lipomas [47, 49–52]. Moreover, as assumed in a recent retrospective analysis based on 77 cases of paratesticular sarcomas, CT and MRI are able to supply prognostically important data for local recurrence concerning tumour size and boundaries; however, the differential diagnosis on the sole basis of imaging can be difficult, with well-differentiated liposarcomas being the most relevant diagnostic challenge [50].

Most paratesticular masses are benign, therefore radical orchiectomy may be avoided: a multiparametric MR protocol including DWI, MTI and DCE could provide important additional information in the assessment of benign nature of paratesticular masses, including cellular angiofibroma the tunica vaginalis, thus preventing unnecessary radical surgery [53].

According to ESUR guidelines, follow-up in testicular microlithiasis is advised if any risk factors are known [54]; in our center, we sometimes use MRI to rule out parenchymal focal abnormalities in patients with grade III microlithiasis [7]. Nevertheless, MRI is unable to depict testicular microlithiasis, but this may facilitate the detection of parenchymal areas with pathological signal or contrast enhancement in affected patients, since the parenchymal echostructural inhomogeneity associated with this condition could not allow malignant lesions to be confidently excluded at US examination.

**Fig. 8:**
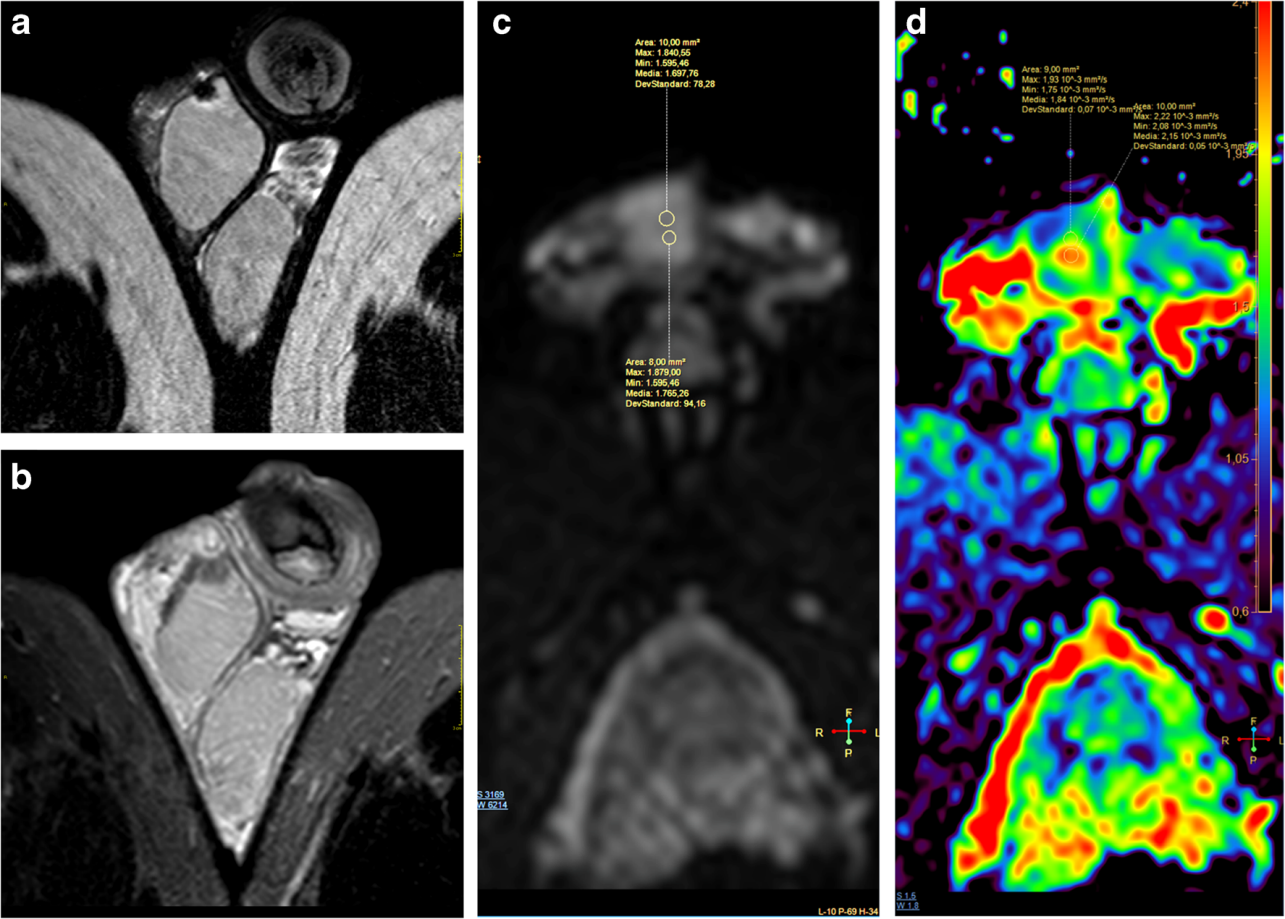
**a**-**d** Hypoechogenic lesion at the site of previous tumour enucleation (Leydig cells tumour). MRI allowed the identification of an area of fibrosis. **a** T2-weighted TSE sequence, coronal view; **b** postcontrast T1-weighted SPIR TSE sequence; c-d. ADC map

MRI proved a useful tool for local staging of testicular neoplasms [29], for testis-sparing surgery planning, if indicated [48], and for the differentiation between fibrosis and local recurrence after nodulectomy (Fig. 8).

In testicular cancer staging after therapy, FDG-PET/CT allows evaluating the presence and vitality of residual disease; nevertheless, false positive results may be reported in seminomas [55], and false negatives in cystic mature teratomas [56].

FDG-PET/CT could be considered a useful adjunct to other imaging modalities in the diagnosis of testicular lymphoma [57] and could depict scrotal extension of peritoneal mesothelioma [58].

### Infarction

Sonographic diagnosis of segmental testicular infarction (STI) is challenging; it’s a rare entity and US appearance is often nonspecific. Anamnestic and clinical information aid orienting the differential diagnosis, but contrast-enhanced MRI is supportive, excluding neoplastic nature of a focal parenchymal lesion [7].

In STI, MRI findings are the following: low signal intensity and a typical rim enhancement after gadolinium administration, the latter related to granulation tissue during the subacute phase [33].

DWI and ADC map of a focal lesion are valuable to exclude or confirm the diagnosis, also through monitoring any change during follow-up MR studies.

In patients with suspected torsion, because the best chance of testicular preservation occurs with expeditious management, it’s recommended an immediate surgical intervention, also without any imaging confirm [59]. Nevertheless, our experience suggests that MRI is useful in selected complex cases, characterized by chronic or intermittent clinical manifestations, including those cases appearing at birth and intrauterine torsion [7]. DWI analysis with ADC values measurement allows identifying testicular torsion without using contrast material [60].

### Varicocele and infertility functional assessment

The role of CT and MR in the evaluation of varicocele is primarily related to the capability to accurately delineate the retroperitoneal anatomy and eventual associated or predisposing conditions [61], notably in those cases of isolated right sided varicocele or monolateral acute varicocele in elderly patients [62].

MRA and retrograde venography depict of a detailed map of the venous spermatic vascularity, useful in selected cases to detect and describe specific vascular abnormalities.

Since varicocele is a frequent condition, often accidentally encountered during US examination, it’s indispensable to obtain functional data to direct exactly therapeutic management. For instance, digital infrared thermography is an imaging modality assessing increased superficial temperature related to varicocele [63].

In a study conducted on 20 cases involving FDG-PET/CT usage for non-neoplastic andrological scopes, investigators found that testicular mean standardized uptake value (SUVmean) showed an inverse correlation with sperm concentration; significant differences were noted between normospermic and oligospermic men, suggesting possible future applications in assisted reproductive technologies and in other andrological or urological fields [64].

DWI with ADC values measurement could be used as a testicular fibrosis index [37].

**Fig. 9:**
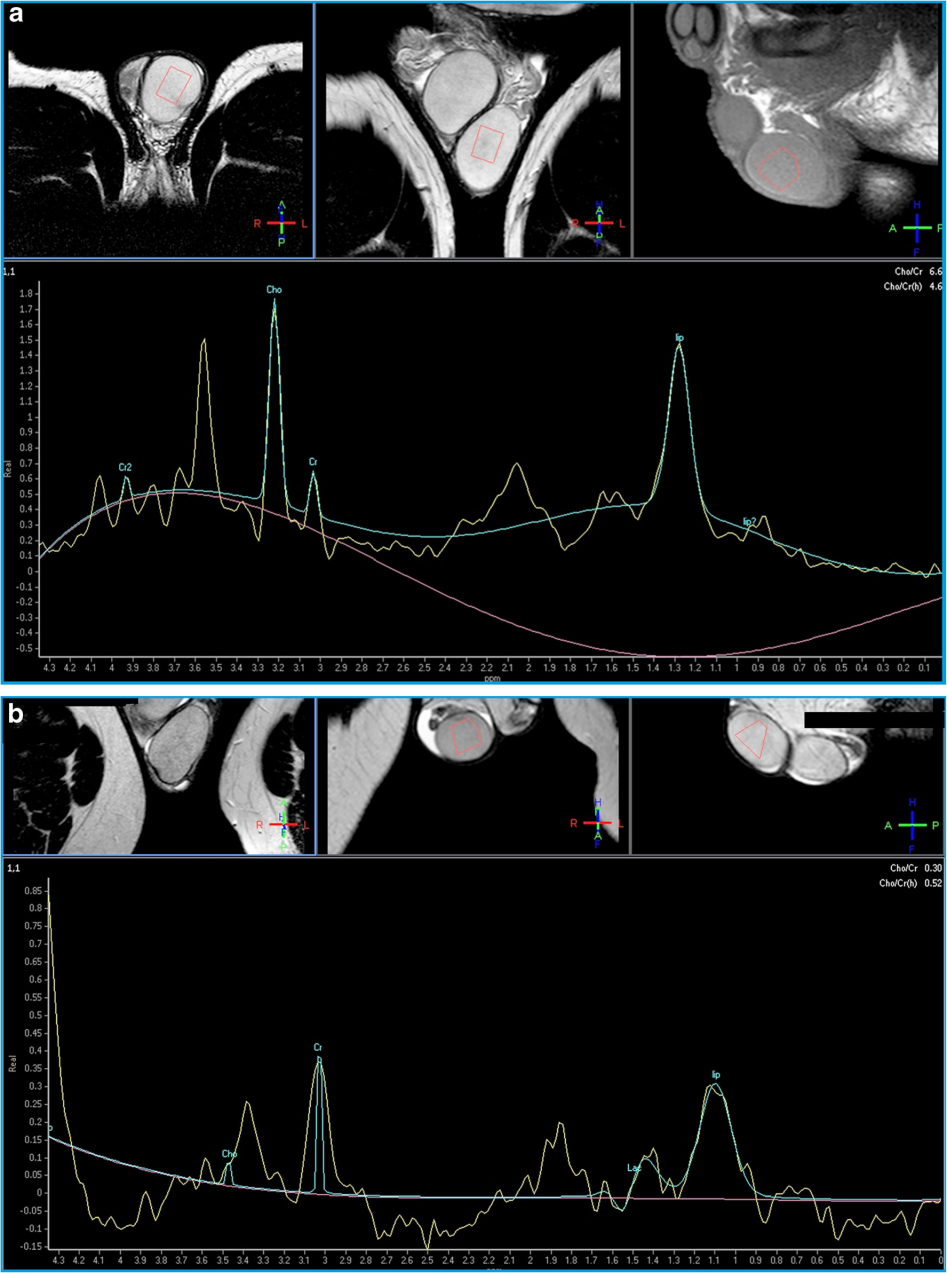
**a**-**b** HRMS1 a. Normal choline peak; **b** altered choline peak related to fertility dysfunction

Our experience with 1HMRS in the evaluation of infertility proved that alterations in choline, creatine, lipids and lactate concentrations in testicular tissue correlate to the level of spermatogenesis (Fig. 9). These values could represent a sign of testicular dysfunction in patients with no evidence of morphological abnormalities [39]. Therefore, 1HMRS may be useful in men presenting with varicocele or other known risk factors for infertility, with no evidence of morphological testicular alterations, to indicate the most appropriate management. This diagnostic approach could be particularly suitable for paediatric patients affected with varicocele, for monitoring testicular function after treatment, or in those cases in which spermiogram cannot be obtained for psychological or religious reasons.

### Congenital abnormalities and scrotal hernia

Accurate diagnosis and appropriate treatment of cryptorchidism lead to the highest chance of proper testicular function in the reproductive capacity terms, and facilitate early detection of malignant degeneration. MRI plays a role in cryptorchid testis investigation: the overall accuracy rate in testes detection is superior for MRI than US, with the first performing much better at locating intra-abdominal testicles [40], also considering its larger field of view. Moreover, undescended testis alterations in echostructure are frequently noted, and MRI allows confidently ruling out superimposed malignancies.

In newborns and paediatric patients with urological or anorectal abnormalities, MRI provides valuable information for preoperative planning, accurately defining even complex anatomic relationships [44].

Finally, MRI is not routinely performed in investigating inguinal-scrotal herniae, but is capable of confirming the diagnosis after clinical and US examination and to reliably depict intestinal loops within the hernia sac [7].

## Conclusions

MRI provides excellent characterization of scrotal focal lesions and valuable depiction of local extent of pathological processes, emerging as the dominant problem-solving imaging modality after a first inconclusive sonographic exam (Table 1).

**Table 1:** Summary of the principal indications for scrotal MRI, as derived from literature analysis

• Correct definition of scrotal masses localisation (testicular vs. extratesticular compartment)
• Characterization of testicular focal lesions after inconclusive findings at ultrasonography
• Local staging of testicular cancer
• Investigation of cryptorchidism (especially for intraabdominal undescended testes)
• Traumatic lesions assessment (selected cases)

CT finds a role in traumatic injuries assessment and in cancer staging together with PET/CT.

Advanced MR techniques, most of all DWI and 1HMRS, are playing a more significant role in scrotal diseases evaluation: they could improve tissue characterization and even provide functional information, for instance allowing early noninvasive identification of patients with spermatogenesis dysfunction.

## References

[1] Kühn AL, Scortegagna E, Nowitzki KM, Kim YH (2016). Ultrasonography of the scrotum in adults. Ultrasonography.

[2] Crawford P, Crop JA (2014). Evaluation of scrotal masses. Am Fam Physician.

[3] Dieckmann K-P, Frey U, Lock G (2013). Contemporary diagnostic work-up of testicular germ cell tumours. Nat Rev Urol.

[4] Studniarek M, Skrobisz-Balandowska K, Modzelewska E (2015). Scrotal imaging. J Ultrasound.

[5] Remer EM, Casalino DD, Arellano RS (2012). ACR appropriateness criteria ® acute onset of scrotal pain--without trauma, without antecedent mass. Ultrasound Q.

[6] Parker RA, Menias CO, Quazi R (2015). MR imaging of the penis and scrotum. Radiographics.

[7] Parenti GC, Feletti F, Brandini F (2009). Imaging of the scrotum: role of MRI. Radiol Med.

[8] Muglia V, Tucci S, Elias J (2002). Magnetic resonance imaging of scrotal diseases: when it makes the difference. Urology.

[9] Baleato-González S, García-Figueiras R, Santiago-Pérez MI (2015). Usefulness of 1H magnetic resonance spectroscopy in human testes: preliminary study. Clin Radiol.

[10] Yamaguchi M, Mitsumori F, Watanabe H (2006). In vivo localized 1H MR spectroscopy of rat testes: stimulated echo acquisition mode (STEAM) combined with short TI inversion recovery (STIR) improves the detection of metabolite signals. Magn Reson Med.

[11] Aaronson DS, Iman R, Walsh TJ (2010). A novel application of 1H magnetic resonance spectroscopy: non-invasive identification of spermatogenesis in men with non-obstructive azoospermia. Hum Reprod.

[12] Firat Uǧraş M, Karakaş HM, Erdem G, Kurus M, Ugras M, Celik T, Kahraman B, Doǧanay SAK (2008). 1H magnetic resonance spectroscopy of the normal testis: preliminary findings. Magn Reson Imaging.

[13] Tsili AC, Giannakis D, Sylakos A (2014). Apparent diffusion coefficient values of normal testis and variations with age. Asian J Androl.

[14] Tsili AC, Ntorkou A, Baltogiannis D (2016). Magnetization transfer imaging of normal and abnormal testis: preliminary results. Eur Radiol.

[15] Tsili AC, Argyropoulou MI, Giannakis D (2012). Diffusion-weighted MR imaging of normal and abnormal scrotum: preliminary results. Asian J Androl.

[16] Howick J, Chalmers I, Glasziou P, et al (2011) The Oxford 2011 levels of evidence. Oxford Centre Evidence-Based Medicine

[17] Kim SH, Park S, Choi SH (2009). The efficacy of magnetic resonance imaging for the diagnosis of testicular rupture: a prospective preliminary study. J Trauma.

[18] Antonini G, Vicini P, Sansalone S (2014). Penile fracture: Penoscrotal approach with degloving of penis after magnetic resonance imaging (MRI). Arch Ital Urol Androl.

[19] Schicho A, Riepl C (2015). Femoral-head dislocation to the scrotum. N Engl J Med.

[20] Boudissa M, Ruatti S, Maisse N (2013). Bilateral testicular dislocation with pelvic ring fracture: a case report and literature review. Orthop Traumatol Surg Res.

[21] Rushambuza RPM (2013). The role of diagnostic CT imaging in the acute assessment of battlefield external genital injuries. J R Army Med Corps.

[22] Imamura T, Horiuchi E (2016). A case of granulomatous Orchitis. Hinyokika Kiyo.

[23] Yoneda A, Fujita F, Tokai H (2010). MRI can determine the adequate area for debridement in the case of Fournier’s gangrene. Int Surg.

[24] Mohrs OK, Thoms H, Egner T (2012). MRI of patients with suspected scrotal or testicular lesions: diagnostic value in daily practice. Am J Roentgenol.

[25] Bulakci M, Tefik T, Kartal MG (2016). Imaging appearances of Paratesticular fibrous Pseudotumor. Polish. J Radiol.

[26] Kim KH, Sung DJ, Han NY (2015). Immunoglobulin G4-related paratesticular fibrous pseudotumor and retroperitoneal fibrosis: a case report. Urol Int.

[27] Tsili AC, Argyropoulou MI, Giannakis D (2010). MRI in the characterization and local staging of testicular neoplasms. Am J Roentgenol.

[28] Manganaro L, Vinci V, Pozza C (2015). A prospective study on contrast-enhanced magnetic resonance imaging of testicular lesions: distinctive features of Leydig cell tumours. Eur Radiol.

[29] Algebally AM, Tantawy HI, Yousef RRH (2015). Advantage of adding diffusion weighted imaging to routine MRI Examinations in the Diagnostics of scrotal lesions. Pol J Radiol.

[30] Tsili AC, Sylakos A, Ntorkou A (2015). Apparent diffusion coefficient values and dynamic contrast enhancement patterns in differentiating seminomas from nonseminomatous testicular neoplasms. Eur J Radiol.

[31] Tsili AC, Ntorkou A, Baltogiannis D (2015). The role of apparent diffusion coefficient values in detecting testicular intraepithelial neoplasia: preliminary results. Eur J Radiol.

[32] Muller J, Schrader AJ, Jentzmik F, Schrader M (2011). Assessment of residual tumours after systemic treatment of metastatic seminoma: (1)(8)F-2-fluoro-2-deoxy-D-glucose positron emission tomography - meta-analysis of diagnostic value. Urologe A.

[33] Parenti GC, Sartoni M, Gaddoni E (2012). Imaging of segmental testicular infarction: our experience and literature review. Radiol Med.

[34] Watanabe Y, Nagayama M, Okumura A (2007). MR imaging of testicular torsion: features of testicular hemorrhagic necrosis and clinical outcomes. J Magn Reson Imaging.

[35] Gulleroglu K (2014). Nutcracker syndrome. World J Nephrol.

[36] Nagappan P, Keene D, Ferrara F (2015). Antegrade venography identifies parallel venous duplications in the majority of adolescents with varicocele. J Urol.

[37] Karakas E, Karakas O, Cullu N (2014). Diffusion-weighted MRI of the testes in patients with varicocele: a preliminary study. Am J Roentgenol.

[38] Gulum M, Cece H, Yeni E (2012). Diffusion-weighted MRI of the testis in hydrocele: a pilot study. Urol Int.

[39] Parenti GC, Albarello F, Campioni P (2016) Role of MR spectroscopy (H1-MRS) of the testis in men with semen analysis altered. Reprod Syst Sex Disord 5: doi: 10.4172/2161-038X.1000182

[40] Krishnaswami S, Fonnesbeck C, Penson D, McPheeters ML (2013). Magnetic resonance imaging for locating nonpalpable undescended testicles: a meta-analysis. Pediatrics.

[41] Kantarci M, Doganay S, Yalcin A, et al (2010) Diagnostic performance of diffusion-weighted mri in the detection of nonpalpable undescended testes: comparison with conventional MRI and surgical findings. Am J Roentgenol 195: doi: 10.2214/AJR.10.4221 10.2214/AJR.10.4221 20858788

[42] Yamada K, Takahata A, Ichijo Y (2014). A case of testicular seminoma in persistent Mullerian duct syndrome with transverse testicular ectopia. Abdom Imaging.

[43] Yildiz A, Yiǧiter M, Oral A, Bakan V (2014). Transverse testicular ectopia. Pediatr Int.

[44] Ziegelmann MJ, Viers BR, Cockerill PA (2017). The utility of magnetic resonance imaging in the evaluation of accessory scrotum in the newborn. Urology.

[45] Wittenberg AF, Tobias T, Rzeszotarski M, Minotti AJ (2006). Sonography of the acute scrotum: the four T’s of testicular imaging. Curr Probl Diagn Radiol.

[46] Karam JA, Baker LA (2007). Focal orchitis presenting as bilateral testicular masses. J Pediatr Urol.

[47] Cassidy FH, Ishioka KM, McMahon CJ (2010). MR imaging of scrotal tumors and pseudotumors. Radiographics.

[48] Tsili AC, Giannakis D, Sylakos A (2014). MR imaging of scrotum. Magn Reson Imaging Clin N Am.

[49] Wolfman DJ, Marko J, Gould CF (2015). Mesenchymal Extratesticular tumors and Tumorlike conditions: from the radiologic pathology archives. Radiographics.

[50] Ap Dafydd D, Messiou C, Thway K (2017). Paratesticular sarcoma: typical presentation, imaging features, and clinical challenges. Urology.

[51] Woodward PJ, Schwab CM, Sesterhenn IA (2003). From the archives of the AFIP: extratesticular scrotal masses: radiologic-pathologic correlation. Radiographics.

[52] Shah T, Abu-Sanad O, Marsh H (2016). Role of magnetic resonance imaging in the early diagnosis of paratesticular rhabdomyosarcoma. Ann R Coll Surg Engl.

[53] Ntorkou AA, Tsili AC, Giannakis D (2016). Magnetic resonance imaging findings of cellular angiofibroma of the tunica vaginalis of the testis: a case report. J Med Case Rep.

[54] Richenberg J, Belfield J, Ramchandani P (2014). Testicular microlithiasis imaging and follow-up: guidelines of the ESUR scrotal imaging subcommittee. Eur Radiol.

[55] Bilen MA, Hariri H, Leon C, et al (2014) Positive FDG-PET/CT scans of a residual seminoma after chemotherapy and radiotherapy: Case report and review of the literature. Clin Genitourin Cancer 12:e147–e150 10.1016/j.clgc.2014.02.006 PMC4104212 24674785

[56] Becherer A (2011) PET in testicular cancer. Humana Press, pp 225–241 10.1007/978-1-61779-062-1_13 21331937

[57] Sidhu P, Lin P, Son H (2014). Testicular fluorine-18 fludeoxyglucose uptake on positron emission tomography CT in patients with lymphoma: clinical significance and management impact. Br J Radiol.

[58] Ozguven S, Aras M, Dede F (2014). Scrotal peritoneal mesothelioma on PET/CT. Clin Nucl Med.

[59] Bowlin PR, Gatti JM, Murphy JP (2017). Pediatric testicular torsion. Surg Clin North Am.

[60] Maki D, Watanabe Y, Nagayama M (2011). Diffusion-weighted magnetic resonance imaging in the detection of testicular torsion: feasibility study. J Magn Reson imaging JMRI.

[61] Belay R, Huang G, Shen J-C, Ko EK (2016). Diagnosis of clinical and subclinical varicocele: how has it evolved?. Asian J Androl.

[62] Nana GR, Basra M, Maudgil DD, Rao AR (2014) Left renal vein thrombosis: a rare cause of acute scrotal pain. BMJ Case Rep 2014:bcr2013202237-bcr2013202237. doi: 10.1136/bcr-2013-202237 10.1136/bcr-2013-202237 PMC3902658 24445848

[63] Kulis T, Knezevic M, Karlovic K (2013). Infrared digital thermography of scrotum in early selection of progressive varicocele. Med Hypotheses.

[64] Dierickx LO, Huyghe E, Nogueira D (2012). Functional testicular evaluation using PET/CT with 18F-fluorodeoxyglucose. Eur J Nucl Med Mol Imaging.

